# Switching Among Biosimilars: A Review of Clinical Evidence

**DOI:** 10.3389/fphar.2022.917814

**Published:** 2022-08-24

**Authors:** Eleonora Allocati, Brian Godman, Marco Gobbi, Silvio Garattini, Rita Banzi

**Affiliations:** ^1^ Center for Health Regulatory Policies, Mario Negri Institute for Pharmacological Research IRCCS, Milan, Italy; ^2^ Department of Pharmacoepidemiology, Strathclyde Institute of Pharmacy and Biomedical Sciences, University of Strathclyde, Glasgow, United Kingdom; ^3^ Centre of Medical and Bio-allied Health Sciences Research, Ajman University, Ajman, United Arab Emirates; ^4^ Division of Public Health Pharmacy and Management, School of Pharmacy, Sefako Makgatho Health Sciences University, Ga-Rankuwa, South Africa; ^5^ Laboratory of Pharmacodynamics and Pharmacokinetics, Mario Negri Institute for Pharmacological Research IRCCS, Milan, Italy; ^6^ Presidency, Mario Negri Institute for Pharmacological Research IRCCS, Milan, Italy

**Keywords:** biosimilar, switch, infliximab, adalimumab, etanercept, therapeutic drug monitoring

## Abstract

Biological medicines have improved patients’ outcomes, but their high costs may limit access. Biosimilars, alternatives that have demonstrated high similarity in terms of quality, safety, and efficacy to an already licensed originator biological product, could increase competition and decrease prices. Given the expanding number of biosimilars, patients may switch from originator to biosimilar or among biosimilars. Randomized trials and observational studies conducted with multiple biosimilars over many disease areas confirmed the safety and efficacy of switching from originator to biosimilar. This study summarizes evidence on switching between biosimilars for which there are concerns to provide future guidance. A systematic search (MEDLINE, Embase, and Cochrane Library) for studies on anti-TNF agents, assessing clinical efficacy and safety of biosimilar-to-biosimilar switch in chronic inflammatory diseases, was performed. We retrieved 320 records and included 19 clinical studies. One study with historical control compared switching between biosimilars to maintenance of the same biosimilar. Ten were controlled cohort studies comparing switching between two biosimilars vs. switching from originator to a biosimilar or vs. multiple switches. Eight were single-arm cohort studies, where participants switched from one biosimilar to another, and the outcomes were compared before and after the switch. Overall, these studies did not highlight significant concerns in switching between biosimilars. Therefore, switching studies seem difficult to perform and unnecessary with the body of evidence suggesting no real problems in practice coupled with stringent regulatory requirements. Monitoring the use of biosimilars in clinical practice could support clinical decision-making, rational use of biological medicines, and help to further realize possible savings.

## Introduction

Biological medicines have appreciably improved the outcomes for patients with immunological diseases including rheumatoid arthritis, psoriatic arthritis, and inflammatory bowel diseases as well as many neoplasms (Alfonso-Cristancho et al., 2017; Nam et al., 2014; Ruyssen-Witrand et al., 2020; Cholapranee et al., 2017; Wilson et al., 2018; da Silva et al., 2018). However, their high costs have limited their use especially in low- and middle-income countries including Central and Eastern European countries (Putrik et al., 2014; Kostić et al., 2017; Baumgart et al., 2019; Gershon et al., 2019; Tubic et al., 2021). The expiration of patents allows the production of biosimilars, alternatives that have demonstrated high similarity in terms of quality, safety, and efficacy to an already licensed originator biological product (Glintborg et al., 2017; Jørgensen et al., 2017; Fiorino et al., 2019b; Meyer et al., 2019; Yang et al., 2019).

Regulatory approval of biosimilars by the European Medicines Agency (EMA) and Food and Drug Administration (FDA) is a rigorous process requiring an extensive comparability exercise based on the assessment of quality, structural, functional, preclinical, and clinical similarity with respect to the originator. While the EMA does not regulate interchangeability between the reference product and biosimilars (European Medicinal Agency and the European commission, 2019), in the USA, the FDA considers the originator and its biosimilars therapeutically interchangeable if the manufacturer has demonstrated no clinically meaningful differences from the reference product (US FDA, 2017).

The expanding number of available biosimilars, and national procurement and reimbursement policies aiming to save costs with ever increasing demands on available resources, inevitably leads to strategies to encourage switching from the originator to less expensive biosimilar(s) in chronic conditions, especially if there are substantial price differences between originators and biosimilars and no differences in effectiveness or safety (Huoponen et al., 2020; Jensen et al., 2020; Moorkens et al., 2020; Godman et al., 2021a; MacBride-Stewart et al., 2021; Vogler et al., 2021). To reduce concerns with switching, many randomized controlled trials (RCTs), real world data in routine clinical care, and systematic reviews have been conducted across countries with multiple biosimilars over many disease areas. These typically show similar effectiveness, safety, and immunogenicity between biosimilars and originators (Danese et al., 2017; Glintborg et al., 2017; Griffiths et al., 2017; Jørgensen et al., 2017; Park et al., 2017; Yoo et al., 2017; Cohen et al., 2018; Matucci-Cerinic et al., 2018; Ratnakumaran et al., 2018; Cohen et al., 2019; Gisondi et al., 2019; Goll et al., 2019; Pegram et al., 2019; Yang et al., 2019; Barbier et al., 2020; Barberio et al., 2021; Bruni et al., 2021; Cingolani et al., 2021; Li et al., 2022). For instance, the NOR-Switch study conducted in Norway provided reassurance that a nonmedical switch from infliximab originator to its biosimilar was not associated with worse outcomes (Jørgensen et al., 2017; Goll et al., 2019). Studies such as these have enhanced the acceptance of biosimilars among clinicians, which is resulting in their more rapid uptake across a number of countries to realize appreciable savings (Matusewicz et al., 2015; Jensen et al., 2020; Godman et al., 2021a; MacBride-Stewart et al., 2021). However, most studies have addressed a single switch from originator to biosimilar with few evaluating multiple or “back and forth” switching between originators and biosimilars or between biosimilars (Blauvelt et al., 2018).

These findings resulted in the World Health Organization (WHO)-in its 2021 Essential Medicine Model List recommending that quality-assured biosimilars should be considered interchangeable (substitution and switching) and eligible for selection and procurement at the country level for national essential medicines lists (World Health Organization, 2021). In addition, competition between biosimilars leading to lower prices will increasingly mean patients potentially being switched between different biosimilars in addition to switching from an originator to a biosimilar.

However, the practice of switching from one biosimilar to another is not presently recommended by a number of scientific societies as well as regulatory agencies as there are still concerns. These include a lack of information regarding potential immunogenicity and the risk of side effects (Cohen et al., 2017; Danese et al., 2017; Position Statement on Biosimilars, 2017; Medicines for Europe, 2019). This may be due to the lack of convincing evidence regarding switching from one biosimilar to another of the same biologic medicine or multiple switches, that is, a treatment sequence including more than one switch between an originator and one or more biosimilars. However, at the same time, regulatory agencies accept multiple changes in the manufacturing of originators without requiring any additional studies even with some changes described as either high or moderate risk (Vezér et al., 2016; Jiménez-Pichardo et al., 2018; Godman et al., 2019).

Consequently, there is a need to further evaluate current evidence regarding switching between biosimilars, sometimes referred to as cross-switching (Mysler et al., 2021), to dispel concerns among key stakeholder groups.

## Methodology

To this aim, we updated the systematic searches launched in October 2021 for the WHO report (Allocati and Gerardi, 2020). We searched MEDLINE, Embase and the Cochrane Library from 2021 to March 2022 for studies on anti-TNF agents assessing clinical efficacy and safety of biosimilar-to-biosimilar switch in chronic inflammatory diseases including Crohn’s disease, ulcerative colitis, rheumatoid arthritis, ankylosing spondylitis, and psoriasis. We included studies on anti-TNF agents as multiple biosimilars have been marked in the European Union for infliximab, adalimumab, and etanercept. We also chose the anti-TNF agents as there have been multiple activities across countries to increase the use of their biosimilars (Moorkens et al., 2017; Jensen et al., 2020; Moorkens et al., 2020; Godman et al., 2021a). For instance, in Norway, price reductions for biosimilar infliximab were already approximately 70% lower than the originator price soon after the launch of the biosimilar (Matusewicz et al., 2015; Godman et al., 2021a). In Denmark, expenditure on adalimumab decreased by 83% following aggressive contracting with multiple biosimilars, with similar expectations for the United Kingdom with estimated savings of over GB£300 million per year (Jensen et al., 2020; Godman et al., 2021a).

We included comparative and single-arm studies. We applied search term for three categories of keywords: “switch/substitution,” “biological medicine/biosimilar,” and “anti-TNF agents” and adapted the search strategy to the three databases (Full search available in the Supplementary Material S1).

One reviewer retrieved the eligible studies and extracted the key information (EA), including the study design, target condition, biological medicine and biosimilars assessed, sample size, and main study outcomes. A second reviewer **(**RB) checked the data extraction. Studies were described narratively.

### Clinical Evidence of Switching Between Biosimilars

We retrieved 189 records from MEDLINE, 141 from Embase, and none from the Cochrane Library. From the screening of titles and abstract, we selected 20 eligible publications (full articles and posters), corresponding to a total of 18 clinical studies. All were included in the analysis. Another publication (abstract) was retrieved by checking the references of other articles and included in the study sample (Bouhnik et al., 2020). Thus, we included a total number of 19 studies. None of them directly compared switching from a biosimilar to another of the same biologic medicine vs. the maintenance of the same biosimilar, either as RCTs or observational studies. These would have been the optimal study designs to assess the efficacy and possible risks of switching between biosimilars (vs. nonswitch), as for the switch between originators to biosimilars. One study, published as poster, compared a group of patients with inflammatory bowel diseases switching from infliximab CT-P13 to SB2 to an historical cohort of patients treated with CT-P13 (Harris et al., 2019). These preliminary data that did not suggest switching had an impact on drug persistence. Ten controlled cohort studies compared switching between two biosimilars vs. switching from originator to a biosimilar or vs. multiple switches, for example, from an originator to biosimilar A to biosimilar B (Lauret et al., 2020; Gall et al., 2021; Hanzel et al., 2021; Khan et al., 2021; Lovero et al., 2021; Luber et al., 2021; Macaluso et al., 2021; Trystram et al., 2021; Lontai et al., 2022; Mazza et al., 2022). Eight were single-arm cohort studies, where participants switched from one biosimilar to another and outcome were compared before and after the switch (Bouhnik et al., 2020; Gisondi et al., 2020; Kiltz et al., 2020; Mott et al., 2021; Peters et al., 2021; Piaserico et al., 2021; Ribaldone et al., 2021; Siakavellas et al., 2021).

Overall, 12 studies adopted a prospective design, six were retrospective, and one (Harris et al., 2019) was a prospective observational study with a retrospective control group. Table 1 shows the details of the included studies and their main results. The total number of participants included in these studies was 3111, with a median number of 133 (range: 36–309). The median follow-up of the included studies was 12 months (range: 4–21 months).

**TABLE 1:** Characteristics of included studies.

First Author (year)	Country	Study design	Indication	N° Pts	Comparison	Main results	Author conclusion
**Infliximab**							
Lovero et al. (2021)	Italy	Cohort study (R)	IBD	36	CT-P13 to SB2 vs. multiple switch	Clinical remission rate, LOR, and AEs: no differences	Switching from CT-P13 to SB2 seems to be safe and effective either in pts with single and multiple switches
Macaluso et al. (2021)	Italy	Cohort study (P)	IBD	276	CT-P13 to SB2 vs. multiple switch vs. IFX originator to SB2	SAEs, n (%)*: CT-P13 to SB2: 11. (25.6) Multiple switches: 4 (16.7)	Safety and effectiveness of IFX SB2 similar to those of IFX originator; switching from originator or CT-P13 (and multiple switches) not dangerous
Hanzel et al. (2021)	The Netherlands	Cohort study (P)	IBD	176	CT-P13 to SB2 vs. multiple switch vs. IFX originator to CT-P13	Clinical remission n (%): CT-P13 to SB2: 55 (69); multiple switch: 58 (84); IFX originator to CT-P13: 25 (93). Discontinuation (HR 95% CI): CT-P13 to SB2: 0.42 (0.16–1.12); multiple switch: 0.39 (0.14–1.11). ADA (%): CT-P13 to SB2: 8.8% (7/80); multiple switch: 5.8% (4/69); IFX originator to CT-P13: none	No significant differences in clinical, CRP, or fecal calprotectin remission at 12 months; lower rates in pts switching from CT-P13 to SB2; multiple switching and switching between biosimilars of IFX seemed effective and safe
Mazza et al. (2022)	Italy	Cohort study (R)	IBD	118	Multiple switch vs. IFX originator to CT-P13	Clinical remission (adjusted OR, 95% CI): 1.3 (0.3–6.2). Total AE n (%): multiple switch 5 (9.6); IFX originator to CT-P13 8 (12.4); discontinuation (adjusted HR, 95% CI) 1.3 (0.3–6.2)	No significant differences in terms of safety and efficacy when comparing double switch with a single switch; data consistent with the safety profile of IFX
Luber et al. (2021)	United Kingdom	Cohort study (P)	IBD	186	CT-P13 to SB2 vs. multiple switch	Disease activity n (%) 1 year: CT-P13 to SB2: 6 (9.5); multiple switch: 1 (1.3). ADA 1 year: none in both arms	Biosimilar switching does not have negative influence in terms of infliximab trough levels and disease activity
Harris et al. (2019)	United Kingdom	Cohort study (P)	IBD	133	CT-P13 to SB2 vs. historic control (no switch)	Disease activity (mean ± SD) week 16–18: Crohn’s disease: 3.15 ± 3.17; Ulcerative colitis: 0.91 ± 1.64	No significant difference in drug levels between historical CT-P13 pts and SB2 pts
Trystram et al. (2021)	France	Cohort study (P)	IBD	204	CT-P13 to SB2 vs. multiple switch	Discontinuation rate n (%): CT-P13 to SB2: 5 (11.6); multiple switch: 7 (6.2). LOR n (%): 17 (10.8) both groups. Clinical remission n (%): CT-P13 to SB2 36/40 (90); multiple switch: 104/113 (92). AEs n (%): CT-P13 to SB2: 13 (31.6); multiple switch: 50 (41.4)	Switching from the originator to CT-P13 and then to SB2 did not impair the effectiveness, immunogenicity or safety of anti-TNF therapy after 54 weeks of follow-up
Bouhnik et al. (2020)	France	Single-arm (R)	IBD	109	IFX (biosimilar or originator) to SB2	LOR n: 19. Discontinuation due to AEs n: 9. Discontinuation due to unspecified reasons n: 16	Switch references or biosimilar IFX to SB2 without loss disease control and no need for dose escalation
Mott et al. (2021)	United Kingdom	Single-arm (P)	IBD	289	CT-P13 to GP1111	LOR n (%): 17 (6)	Proportion of pts who discontinued due to LOR consistent with historical norm; switching between biosimilar IFX is safe and effective
Siakavellas et al. (2021)	United Kingdom	Single-arm (P)	IBD	246	CT-P13 to GP1111	ADA n (%): 5 (2). Discontinuation rate n (%): 10 (3.7). LOR n (%): 5 (2)	Single and multiple biosimilar IFX switching is safe with no negative effects in clinical outcomes at 6 months
Lauret et al. (2020)	France	Cohort study (P)	CID	309	CT-P13 to SB2 vs. multiple switch	ADA n (%) 3 years: CT-P13 to SB2: 11 (25); multiple switch: 20 (8.5). Discontinuation rate n (%) 3 years: CT-P13 to SB2: 15 (34); multiple switch: 44 (16.6). Retention rate n (%) 3 years: CT-P13 to SB2: 29 (66); multiple switch: 155 (58)	Demonstration of comparable immunization rate regardless of the number of biosimilars received; successive use of two biosimilars did not increase risk of immunogenicity
Peters et al. (2021)	The Netherlands	Single-arm (R)	Sarcoidosis	86	IFX originator or CT-P13 to SB2	Discontinuation: none; AE n (%): 5 (6.3); ADA (assessed in 7 pts): none	None of the pts discontinued six months after switching from originator to a biosimilar; IFX trough levels before and after switch did not significantly changed compared with trough levels at baseline
Gisondi et al. (2020)	Italy	Single-arm (P)	Psoriasis	96	Multiple switch	Mean PASI: no change. LOR n (%): 7 (7.3). AE n (%): 3 (3.1)	Switch not associated with significant change in the mean PASI and LOR
Khan et al. (2021)	United States	Cohort study (R)	CIRD	271	Multiple switch vs. IFX originator to SB2	Discontinuation rate n (%): multiple switch: 30 (17.6); IFX originator to SB2: 9 (8.9). LOR n (%): multiple switch: 15 (8.8); IFX originator to SB2: 9 (8.9). Pts not in remission n (%): multiple switch: 16 (9.4); IFX originator to SB2: 12 (11.9)	Pts with stable disease activity at baseline, there was no statistically significant difference in efficacy or safety when switching from IFX to SB2 or multiple switch
**Adalimumab**							
Ribaldone et al. (2021)	Italy	Single-arm (P)	CID	68	ABP501 to SB5	Success rate (clinical remission) n (%): 50 (82) discontinuation n (%): 7 (11.5). AE n (%): 7 (11.5)	Switching between biosimilars is safe and effective; switch not recommended if positive CRP is found at the time of switching
Lontai et al. (2022)	Hungary	Cohort study (P)	IBD	246	ADM bio 1 to ADM bio 2 vs. ADM originator to ADM bio	Clinical remission % (week 20–24): bio1 to bio2: 77.6; originator to bio: 85	No differences in pts who switched from originator to biosimilar or between biosimilars
Gall et al. (2021)	N/A	Cohort study (P)	CIRD	90	ADM bio 1 to ADM bio 2 vs. multiple switch	No differences in disease characteristics nor in satisfaction with care	No differences in disease characteristics or in satisfaction with care
**Etanercept**							
Kiltz et al. (2020)	Germany	Single-arm (R)	CIRD	100	SB4 to GP2015	DAS28 (RA) mean ± SD: 3.0 (1.4); DAS28 (PsA) mean ± SD: 3.6 (2.6); BASDAI (axSpA) mean ± SD: 4.3 (2.4); Discontinuation n: 7 pts; AEs n: 8 pts	Retention rate after multiple switches about 90%; no major changes in disease activity and function
Piaserico et al. (2021)	Italy	Single-arm (P)	Psoriasis	72	Multiple switch (originator to SB4 to GP 2015)	LOR n: 3 pts. No treatment-emergent SAEs reported	Switching from SB4 to GP2015 is both safe and effective

*Results of the groups in which patients switch between biosimilars.

ADA, antidrug antibodies; ADM, adalimumab; AEs, adverse events; axSpA, axial spondyloarthritis; BASDAI, Bath Ankylosing Spondylitis Disease Activity; CI, confidence interval; CI(R)D, chronic inflammatory (rheumatic) diseases; CPR, C-reactive protein; DAS28, disease activity score; IFX, infliximab; LOR, loss of response; multiple switch, switch from originator to one biosimilar and then to another; P, prospective; PASI, psoriasis area severity index; PsA, psoriatic arthritis; Pts, patients R, retrospective; SAE, severe adverse events; SD, standard deviation.

**FIGURE 1:**
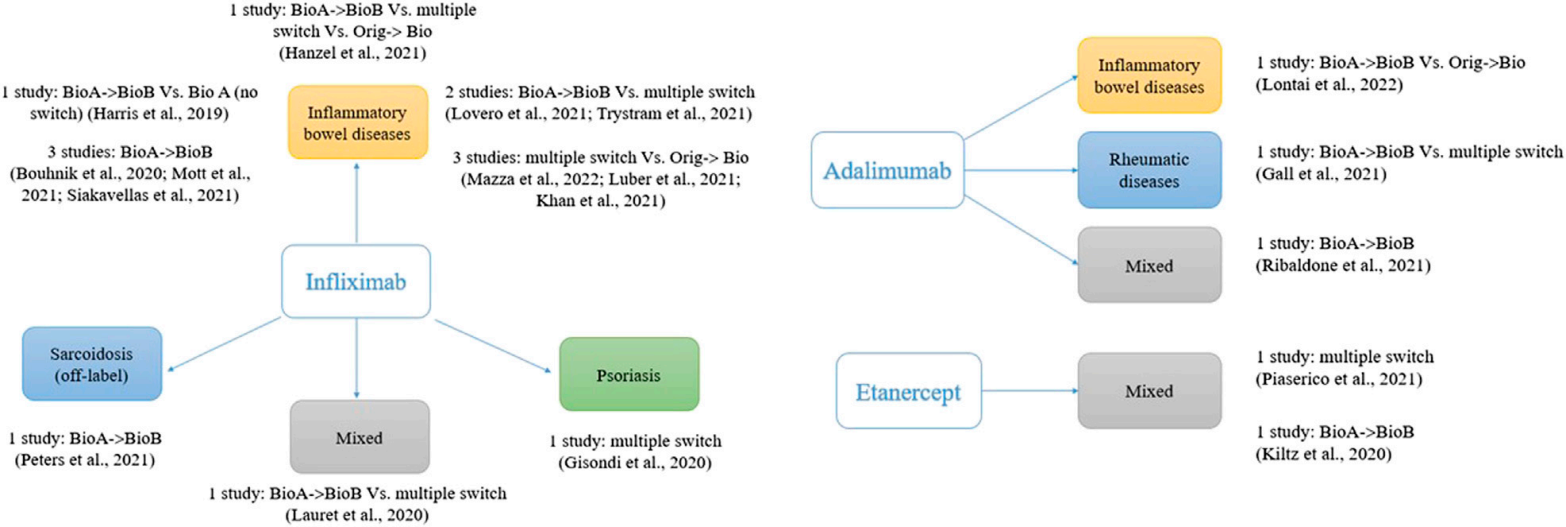
Studies assessing switch between biosimilars of anti-TNF; one study with five comparisons not included (Macaluso et al., 2021).

As shown in Figure 1, most of the studies (74%, 14 out of 19) involved infliximab (originator and the biosimilars CT-P13 and SB2). This is likely to be due to the immunogenicity concerns regarding infliximab, which is a chimeric human/murine IgG1 monoclonal antibody (mAb) able to induce the production of human anti-infliximab antibodies (Pecoraro et al., 2017). Moreover, infliximab is among the most prescribed biosimilars worldwide.

Most of the studies (63%, 12 out of 19) assessed anti-TNF for the management of inflammatory bowel diseases (IBD), ulcerative colitis, or Crohn’s disease in clinical practice setting. The first biosimilar for the treatment of IBD was introduced in 2013, and by the end of March 2022, 14 anti-TNF alpha biosimilar active principles (three for infliximab, eight for adalimumab, and three for etanercept) have been licensed by the EMA (European Medicinal Agency Biosimilars, 2022). The pivotal studies supporting the authorization of these biosimilars all included participants with chronic conditions other than inflammatory bowel diseases, but they were licensed for these indications following the principle of extrapolation of indications (Allocati and Gerardi, 2020; European Medicinal Agency Biosimilars, 2022).

This approach caused some reluctance among gastroenterologists regarding the use of biosimilars, which resulted in the instigation of several clinical studies with biosimilars for IBD in different countries and settings (Jørgensen et al., 2017; Ratnakumaran et al., 2018; Ye et al., 2019; Iniesta Navalón et al., 2021; Schreiber et al., 2021). These studies, coupled with the real-life clinical experiences, have progressively changed the point of view of physicians (Fiorino et al., 2019a; Bhat and Qazi, 2021).

It is worth noting that one study analyzed the switching between two infliximab biosimilars in patients with sarcoidosis, an inflammatory disorder characterized by a heightened granulomatous immune response (Peters et al., 2021). Infliximab is used off-label to treat this condition, as multiple studies demonstrated a clinical improvement, possibly because of the cytokine TNF-α role in the inflammatory process and granuloma formation.

In terms of outcome, all the included studies evaluated whether the switch between biosimilars impacted on the safety and efficacy of anti-TNF agents. Safety was typically measured as the frequency of adverse events and discontinuations, while efficacy was assessed by measuring clinical responses or worsening of the disease, steroid-free clinical remission, or loss of response, through standard metrics applied to the different diseases. For instance, serum C-reactive protein levels were measured in inflammatory disease and American College of Rheumatology (ACR) criteria used in rheumatic disorders. Less than a third of the included studies (26%, 5 out of 19) specifically addressed the impact on immunogenicity, by measuring infliximab trough levels and antidrug antibodies using ELISA assay (Lauret et al., 2020; Hanzel et al., 2021; Luber et al., 2021; Peters et al., 2021; Trystram et al., 2021).

Overall, these studies suggest that switching from biosimilar (infliximab, adalimumab, or etanercept) to another biosimilar of the same medicinal biologic medicine in patients with chronic inflammatory diseases is safe and effective in terms of disease activity, remission rate, loss of response, adverse events, and immunogenicity (when analyzed). Similar conclusion can be drawn from studies assessing multiple switches, that is, studies in which patients already on treatment with the originator are switched to one biosimilar and then to another one. None of the studies assessing immunogenicity demonstrated that switching between biosimilars leads to a change in the immune response, with similar antidrug antibodies trough levels either soon after switching or after longer follow-up (Table 1).

### Discussion and Potential Next Steps

The lack of studies that directly compared switching from a biosimilar to another of the same biologic medicine vs. the maintenance of the same biosimilar could lead to a call for further (high-quality) studies to dispel concerns about switching between biosimilars. However, a serious reflection on the relevance of this research is needed in light of our findings. It is true that the medical community have expressed some reservations about interchangeability and switching, with immunogenicity frequently raised as main concern. However, clinical studies to date that have focused on switching between the originator reference product and biosimilars have been able to reassure the prescribers through confirming substantial equivalence. Moreover, the increasing number of biosimilars available on the market makes it extremely challenging to conduct standard parallel trials comparing all the possible sequence combinations. This heterogeneity is clear observing the fragmentation of the treatment sequences (Figure 1). The analysis is limited to anti-TNF drugs for chronic inflammatory diseases. Although we cannot exclude different scenarios, it is likely that similar reflections apply to other biologics or disease areas.

Switching is typically triggered by nonmedical decisions including cost or procurement issues given the typically high and growing cost of new biological medicines especially in disease areas such as cancer and orphan diseases (Luzzatto et al., 2018; Godman et al., 2021b; Mysler et al., 2021). Hurdles in the development of biosimilars including the request for studies demonstrating their efficacy and safety after switching can appear disproportionate and may discourage companies from developing biosimilars, which will be detrimental to key stakeholder groups in the future. The greater the number of companies that develop biosimilars, the greater the potential price discounts, which is the ultimate goal of health authorities with increasing pressures on their budgets.

In the rapidly evolving scenario of currently available biosimilars for inflammatory chronic diseases and given that RCTs are unfeasible, disease registries and prescription monitoring may be feasible alternatives with providing relevant information for physicians in everyday practice. Data collected during clinical practice in well-conducted observational studies (the so-called real-world data) can provide relevant and valuable evidence, complementary to those derived from RCTs, on the effectiveness and safety of biosimilars across multiple indications and treatment setting. Moreover, therapeutic drug and immunogenicity monitoring (TDIM), that is, the measurement of drug and antidrug antibodies to individualize treatment strategy, has been proposed as a method to maximize efficacy, safety, and cost-effectiveness of anti-TNF therapy (Bloem et al., 2017; Medina et al., 2017; Ricciuto et al., 2018; Ma et al., 2019; Papamichael et al., 2019). This is particularly important when switching patients from originators to considerably less expensive biosimilars and when there are concerns with the effectiveness in practice. The envisaged availability and convenience of TDIM may help ascertain the rationale for any decrease in effectiveness with switching and avoid automatic switch back to the originator in patients with a loss of response, approximately 25–30% patients (Qiu et al., 2017). Recently, a RCT conducted among 20 Norwegian hospitals showed that proactive TDIM during maintenance therapy with infliximab (the originator or a biosimilar product) was more likely to lead to sustained disease control in patients with immune-mediated inflammatory diseases (Syversen et al., 2021; Wallace and Sparks, 2021). However, proactive monitoring is currently not routinely offered to patients treated with biological medicines across countries. Despite the promising results of the Norwegian trial, other studies assessing the clinical utility of TDIM over empirical decisions have reported conflicting results (Ricciuto et al., 2018; Borren et al., 2021). The variety of analytical methods and thresholds may be one of the key drivers of these contradictions. Various immunoassay approaches have been used to detect and quantify ADA (Beeg et al., 2021), and the comparison of different techniques highlighted different results in terms of ADA titers (Steenholdt et al., 2013). As regards ELISA, that is, the most common assay, a diagnostic guidance of NICE, comparing commercial and in house ELISA kits, raised concerns on their analytical performance (NICE, 2016). More recent data suggested that ELISA can result in an underestimation, or even the lack of detection, of ADA (Beeg et al., 2021). A recent survey of 80 studies showed that the proportion of ADA-positive patients varies widely, from 4.8 to 79%, depending on the assay (Gorovits et al., 2018). These data call for unified and validated analytical approaches to increase the reliability of ADA measurements during treatment with anti-TNF agents.

While some clinical guidance recommends TDIM when patients loss response to treatment (reactive monitoring) (Feuerstein et al., 2017; Gomollón et al., 2017), it has not widely been adopted and currently not typically reimbursed by national health services, as seen, for example, in Italy. If the usefulness of TDIM to support clinical decisions, and thereby improving patients’ outcomes and the rational use of biologic agents, can be confirmed, it may become a key tool for the management of the increasing number of patients undergoing switching between originators and biosimilars as well as between biosimilars.

Routine patient monitoring may also have a positive impact on discontinuation or adverse events from biosimilars where these are caused by patients’ negative perception of biosimilars or any change in therapy, the so-called nocebo effect. In particular, the emergence of side effects after switching and their resolution after reverting to the formulation previously prescribed (originator or another biosimilar) may have been a result of the nocebo effect (Odinet et al., 2018; Rezk and Pieper, 2018; Colloca et al., 2019).

Patient information remains essential to strengthen their relationship with the doctor and to accept biosimilars, including switching between biosimilars, and TDIM can help in this respect along with general patient information.

### Final Remarks

There is a need to increase physicians’ and patients’ confidence in biosimilar medicines, including switching between biosimilars, to increase the availability and use of biological medicines especially where there are issues of affordability.

The findings from the 19 identified studies show that whether switching for the first or second time, there was no significant difference in the efficacy and safety of biosimilars, particularly if patients are in remission at the time of the switch. This is similar to the multiple studies that have shown similar effectiveness, safety, and immunogenicity between biosimilars and originators (Danese et al., 2017; Glintborg et al., 2017; Griffiths et al., 2017; Jørgensen et al., 2017; Park et al., 2017; Yoo et al., 2017; Cohen et al., 2018; Matucci-Cerinic et al., 2018; Ratnakumaran et al., 2018; Cohen et al., 2019; Gisondi et al., 2019; Goll et al., 2019; Pegram et al., 2019; Yang et al., 2019; Barbier et al., 2020; Barberio et al., 2021; Bruni et al., 2021; Cingolani et al., 2021; Li et al., 2022). In addition to data supporting biosimilarity at the time of approval, these data should reassure professional societies and patient groups who strongly advocate that any decision to exchange an originator with a biosimilar should remain the responsibility of the physicians in consultation with their patients.

Potential savings, enhanced by increasing competition between biosimilar manufacturers, with competition potentially further increased by WHO prequalification scheme (Davio, 2019; Hagen, 2020; Godman et al., 2021a; Haque et al., 2021), can subsequently be used to enhance the number of patients receiving biologicals to manage their disease (Dutta et al., 2020).

In view of our findings, healthcare professional expectations for routine switching studies now seem unnecessary with the growing body of evidence suggesting no real problems in practice coupled with stringent regulatory requirements. Increased monitoring of patients prescribed biosimilars in clinical practice through increased use of TDIM that could offer an additional tool to support interchangeability and help to further realize possible savings.

## Data Availability

The raw data supporting the conclusion of this article will be made available by the authors, without undue reservation.
