# Supplementary material for: Switching Among Biosimilars: A Review of Clinical Evidence
Source: Front Pharmacol. 2022 Aug 24;13:917814. doi: 10.3389/fphar.2022.917814 (PMC9449694; doi:10.3389/fphar.2022.917814)
Supplement: Supplementary file 1 [file DataSheet1.docx]

**Search strategies - SWITCH anti-TNF alfa**

**PUBMED 04 FEBRUARY 2022**

(((((((((((((("Drug Substitution"[Mesh] OR "Switch"[All] OR "switching"[All] OR "switched"[All] OR "switches"[All] OR "substitute"[All] OR "substitutes"[All] OR "substitution"[All] OR "substituted"[All] OR "substituting"[All] OR "interchange"[All] OR "interchanges"[All] OR "interchanged"[All] OR "interchanging"[All] OR "interchangeability"[All] OR "interchangeable"[All] OR "inter-change"[All] OR "inter-changes"[All] OR "inter-changed"[All] OR "inter-changing"[All] OR "inter-changeability"[All] OR "inter-changeable"[All] OR "inter change"[All] OR "inter changes"[All] OR "inter changed"[All] OR "inter changing"[All] OR "inter changeability"[All] OR "inter changeable"[All] OR "switchability"[All])))))) AND ((((("Biosimilar pharmaceuticals"[Mesh] OR "biosimilar"[All] OR "biosimilars"[All] OR "biosimilarity"[All] OR "similar biological medicine"[All] OR "similar biological medicines"[All] OR "similar biological medicinal product"[All] OR "similar biological medicinal products"[All] OR "follow on biologic"[All] OR "follow-on biologic"[All] OR "follow on biologics"[All] OR "follow-on biologics"[All] OR "Subsequent entry biologic"[All] OR "Subsequent-entry biologic"[All] OR "Subsequent entry biologics"[All] OR "Subsequent-entry biologics"[All] OR "follow on biological"[All] OR "follow-on biological"[All] OR "follow on biologicals"[All] OR "follow-on biologicals"[All] OR "Subsequent entry biological"[All] OR "Subsequent-entry biological"[All] OR "Subsequent entry biologicals"[All] OR "Subsequent-entry biologicals"[All]))))))))) AND (((((((adalimumab) OR "Adalimumab"[Mesh]) OR humira)) OR (((etanercept) OR "Etanercept"[Mesh]) OR enbrel)) OR ((("Infliximab"[Mesh] OR "Infliximab"[All] OR "Jaximab"[All] OR "Remicade"[All] OR "SCH 215596"[All] OR "SCH215596"[All] OR "SCH-215596"[All] OR "TA 650"[All] OR "TA-650"[All] OR "TA650"[All])))))

**n. 189 results**

**EMBASE 06 MARCH 2022**

('drug substitution'/exp OR 'drug substitution' OR 'switch' OR 'switching' OR 'interchange' OR 'interchangeability' OR 'switchability') AND ('biosimilar agent' OR 'biosimilar drug' OR 'follow on biological') AND (infliximab OR 'adalimumab' OR 'etanercept' OR 'infliximab'/exp/mj OR 'adalimumab'/exp/mj OR 'etanercept'/exp/mj) AND ('inflammatory bowel disease' OR 'crohn disease' OR 'rheumatic disease' OR 'psoriasis' OR 'inflammatory bowel disease'/exp/mj OR 'rheumatic disease'/exp/mj OR 'psoriasis'/exp/mj) AND [embase]/lim

**n. 141 results**

**COCHRANE LIBRARY 06 MARCH 2022**

#1 (“Drug Substitution” OR “Switch” OR “switching” OR “switched” OR “switches” OR “substitute” OR “substitutes” OR “substitution” OR “substituted” OR “substituting” OR “interchange” OR “interchanges” OR “interchanged” OR “interchanging” OR “interchangeability” OR “interchangeable” OR “inter-change” OR “inter-changes” OR “inter-changed” OR “inter-changing” OR “inter-changeability” OR “inter-changeable” OR “inter change” OR “inter changes” OR “inter changed” OR “inter changing” OR “inter changeability” OR “inter changeable” OR “switchability”) 30044

#2 MeSH descriptor: [Drug Substitution] explode all trees 378

#3 #1 or #2 30044

#4 (“Biosimilar pharmaceuticals" OR "biosimilar" OR "biosimilars" OR “biosimilarity” OR "similar biological medicine" OR "similar biological medicines" OR "similar biological medicinal product" OR "similar biological medicinal products" OR “follow on biologic" OR “follow on biologics” OR “Subsequent entry biological” OR “Subsequent-entry biological” OR “Subsequent entry biologicals” OR “Subsequent-entry biologicals”) 1089

#5 MeSH descriptor: [Biosimilar Pharmaceuticals] explode all trees 164

#6 #4 or #5 1089

#7 (“Infliximab” OR “Infliximab” OR “Jaximab” OR “Remicade” OR “SCH 215596” OR “SCH215596” OR “SCH-215596” OR “TA 650” OR “TA-650” OR “TA650”) 2574

#8 MeSH descriptor: [Infliximab] explode all trees 722

#9 #7 OR #8 2574

#10 ("adalimumab"):ti,ab,kw OR ("Humira"):ti,ab,kw 3004

#11 MeSH descriptor: [Adalimumab] explode all trees 744

#12 #10 OR #11 3004

#13 ("etanercept"):ti,ab,kw OR ("Enbrel"):ti,ab,kw 2184

#14 MeSH descriptor: [Etanercept] explode all trees 758

#15 #13 OR #14 2184

#16 #9 OR #12 OR #15 6563

#17 #3 AND #6 AND #16 130

#18 "accession number" near pubmed 685193

#19 "accession number" near EMBASE 569507

#20 #18 OR #19 1035176

#21 #17 NOT #20 with Cochrane Library publication date After Oct 2020 **0**
